# Dynamic Assignment and Maintenance of Positional Identity in the Ventral Neural Tube by the Morphogen Sonic Hedgehog

**DOI:** 10.1371/journal.pbio.1000382

**Published:** 2010-06-01

**Authors:** Eric Dessaud, Vanessa Ribes, Nikolaos Balaskas, Lin Lin Yang, Alessandra Pierani, Anna Kicheva, Bennett G. Novitch, James Briscoe, Noriaki Sasai

**Affiliations:** 1 Developmental Neurobiology, MRC-National Institute for Medical Research, London, United Kingdom; 2 Department of Neurobiology, Broad Center of Regenerative Medicine and Stem Cell Research, David Geffen School of Medicine at UCLA, Los Angeles, California, United States of America; 3 Institut Jacques Monod, Université Paris Diderot, Program of Development and Neurobiology, Paris, France; Stanford University, United States of America

## Abstract

Morphogens are secreted signalling molecules that act in a graded manner to control the pattern of cellular differentiation in developing tissues. An example is Sonic hedgehog (Shh), which acts in several developing vertebrate tissues, including the central nervous system, to provide positional information during embryonic patterning. Here we address how Shh signalling assigns the positional identities of distinct neuronal subtype progenitors throughout the ventral neural tube. Assays of intracellular signal transduction and gene expression indicate that the duration as well as level of signalling is critical for morphogen interpretation. Progenitors of the ventral neuronal subtypes are established sequentially, with progressively more ventral identities requiring correspondingly higher levels and longer periods of Shh signalling. Moreover, cells remain sensitive to changes in Shh signalling for an extended time, reverting to antecedent identities if signalling levels fall below a threshold. Thus, the duration of signalling is important not only for the assignment but also for the refinement and maintenance of positional identity. Together the data suggest a dynamic model for ventral neural tube patterning in which positional information corresponds to the time integral of Shh signalling. This suggests an alternative to conventional models of morphogen action that rely solely on the level of signalling.

## Abstract 2

During development of the vertebrate neural tube, cells acquire their positional identity from not only the spatial level of the Sonic Hedgehog signaling gradient, but also the temporal duration.

## Author Summary

In many developing tissues, the pattern in which cell types are generated depends on secreted factors called morphogens. These signalling molecules are produced in specific locations and at specific concentrations, thereby forming concentration gradients. Different target genes are induced at specific distances from the source of the morphogen, and therefore the spatial pattern of gene expression correlates with this concentration gradient. In this study, we examined how cells respond to a morphogen gradient to produce the appropriate pattern of cellular differentiation. We focused on the morphogen Sonic Hedgehog (Shh), which specifies the pattern of different types of neurons in the ventral regions of the neural tube (the embryo's precursor to the central nervous system). We show that in addition to the concentration of Shh, the duration of Shh signalling also contributes to the patterning of the ventral neural tube. A consequence of this is that the genes defining different cellular identities are expressed in a characteristic temporal progression. In addition, sustained Shh signalling is required for more ventral cell types; otherwise they revert to their previous cellular identity. Together these results indicate that dynamic and sustained signalling by Shh is required for the patterning of the ventral neural tube, challenging conventional models of morphogen action that rely solely on the concentration of signal perceived by cells at specific positions in the morphogen gradient.

## Introduction

A defining feature of embryogenesis is the specification of a large variety of cell types in stereotypical spatial and temporal patterns. A common mechanism by which this is achieved involves the deployment of morphogens [1],[2],[3]. These secreted molecules are proposed to establish signalling gradients within developing tissue that provide the positional information that guides the pattern of gene expression and cellular differentiation. Of central importance, therefore, is to understand the nature of the positional information produced by the morphogen.

Several families of secreted proteins, including members of the Hedgehog (Hh), Transforming Growth Factor beta (TGF-β), and Wingless (Wnt) families, operate as morphogens during embryonic development [2],[4]. One example where progress has been made in understanding the mechanism of morphogen action is Sonic Hedgehog (Shh) signalling in the vertebrate central nervous system [5],[6],[7]. Shh is produced from the ventral midline of the neural tube and underlying notochord [8],[9],[10],[11],[12],[13] and spreads to form a ventral-to-dorsal gradient [14]. Within responding cells, Shh signalling regulates the activity of Gli transcription factors (Gli1, 2, and 3) to produce a net increase in their transcriptional activator function [15],[16],[17],[18],[19]. This, in turn, controls the expression of a set of transcription factors in ventral progenitor cells that subdivide the neuroepithelium into five molecularly distinct domains arrayed along the dorsal-ventral (DV) axis [5],[20]. Each progenitor domain generates one of five different neuronal subtypes; from dorsal to ventral, the domains are termed p0, p1, p2, pMN, and p3 and generate V0, V1, V2 neurons, motor neurons (MNs), and V3 neurons, respectively [5],[20],[21].

The two most ventral neural progenitor domains, p3 and pMN, are defined by the expression of the transcription factors Nkx2.2 and Olig2, respectively [22],[23]. In response to Shh signalling, Olig2 is expressed first, in a small group of ventral neural progenitors [14],[24]. Its expression then gradually expands dorsally. As this happens, the ventral cells that originally expressed Olig2 induce Nkx2.2 expression and downregulate Olig2. Nkx2.2 expression then also expands dorsally, downregulating Olig2 in its wake [22],[23],[24], however the dorsal expansion of Nkx2.2 is more limited than Olig2. The end result of the sequential induction and expansion of these two factors is two adjacent but spatially distinct progenitor domains: Olig2 expressing pMN progenitors located dorsal to Nkx2.2 expressing p3 cells. Importantly, the order of appearance and the final position of these progenitor domains correspond to their requirement for increasing concentrations of Shh [21],[24],[25]—the more ventrally located progenitor domain emerges later and requires higher Shh concentrations. One explanation for this, which would accord with the conventional model for morphogen interpretation [1],[3], is that Shh concentration increases over time [14]. As a consequence of this, the threshold concentration for Nkx2.2 activation would be reached later than that of Olig2 [14]. However, our previous work suggested a more complex mechanism of morphogen gradient interpretation in which the signalling pathway does not linearly transduce Shh concentrations required for Olig2 and Nkx2.2 induction [24].

In this non-canonical model of morphogen interpretation, not only the level of intracellular signalling but also its duration plays a crucial role. In vitro experiments indicate that signal transduction is not linearly proportional to Shh concentration at the concentrations of Shh that induce Nkx2.2 and Olig2 [24]. In fact, initially in cells exposed to these concentrations of Shh, signal transduction is saturated resulting in similarly high levels of intracellular Gli activity. However, cells gradually adapt their response to Shh so that they become increasingly less sensitive to the ligand. This means that the time for which intracellular signalling is maintained above a particular threshold is proportional to the extracellular concentration of Shh. Consequently, the concentration of Shh that induces Nkx2.2 maintains high levels of intracellular signalling for a longer period of time than the lower, Olig2-inducing concentration. The gradual adaptation of cells to Shh is controlled, at least in part, by the Shh mediated upregulation of Ptc1, the receptor for Shh that is also a negative regulator of the pathway [26],[27],[28],[29],[30],[31]. High Shh concentrations bind to and suppress more Ptc1 than lower Shh concentrations, thereby sustaining intracellular signal transduction for longer [32],[33]. Importantly, a longer period of signalling is required for the induction of Nkx2.2 than for Olig2. Together, therefore, the data indicate that the differential response to the concentrations of Shh that induce Nkx2.2 or Olig2 is a function of the duration of intracellular signalling rather than the levels of signalling. Hence, at these concentrations, increasing the extracellular concentration of Shh results in an increase in the duration of intracellular signalling rather than a change in the level of signalling. We termed this mechanism “temporal adaptation” [24].

Whether this mechanism is relevant for the provision of positional information throughout the concentration range of Shh in the ventral neural tube remains an open question. Moreover, the kinetics of Shh signalling that assign positional identity are unclear, as is the length of time that Shh signalling is required to maintain positional identity once established. To address these issues we first focused on the specification of V0, V1, and V2 neurons, which arise from progenitors located dorsal to the pMN and require lower concentrations of Shh than MNs and V3 neurons [34]. We show that these concentrations of Shh do not saturate the signal transduction pathway and Gli activity levels are proportional to Shh concentration. Nevertheless, we provide evidence that the duration of Shh signalling plays an important role in the specification of V0–V2 neurons, such that increasing durations of signalling promote the generation of more ventral identities. Moreover, we provide in vivo evidence that the assignment of progenitor identity in this region of the neural tube is progressive. Together these data indicate that the duration of Shh signalling is important for all ventral progenitors to acquire their correct positional identity. We further show that sustained Shh signalling is required to maintain appropriate progenitor pattern in the ventral neural tube even after positional identity has been induced. Progenitors revert to antecedent identities if signalling is interrupted. Thus the allocation of cell identity in the ventral neural tube appears more plastic than for other well-described morphogen patterned tissues and we discuss the implications for models of tissue patterning by morphogen gradients. Together, the data suggest a model for ventral neural tube patterning in which positional identity of progenitors is dynamic and determined by the level and duration—the time integral—of Shh signalling.

## Results

### Distinct Temporal Profiles of Gli Activity Are Generated by Different Shh Concentrations

**Figure 1:**
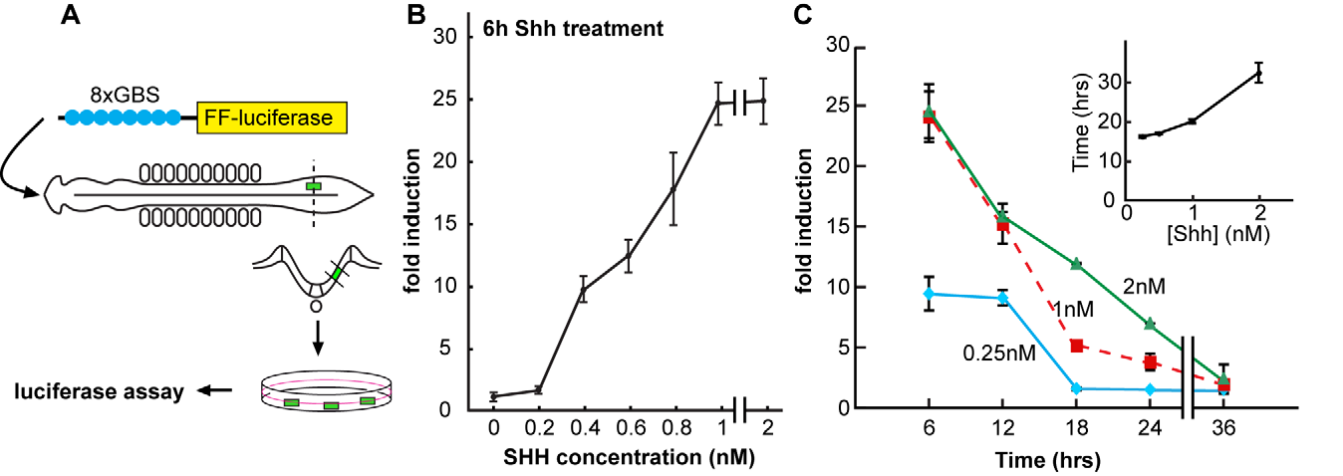
Temporal profile of Gli activity in neural cells exposed to different concentrations of Shh. (A) Experimental scheme. The reporter plasmid, which carries the coding sequence of the firefly luciferase gene (FF-luciferase) driven by octameric Gli-binding sites (8xGBS), was introduced with a normalization plasmid, containing Renilla luciferase, into HH st.10 chick embryos by in ovo electroporation. [i] Explants were removed and cultured ex vivo in the presence of different concentrations of recombinant Shh. Dual luciferase assays were performed at appropriate time points. (B) Gli activity, measured with GBS-Luc, in neural cells exposed to the indicated concentrations of Shh (nM) for 6 h. Graph shows the fold induction of Gli activity in response to Shh compared to the level of Gli activity in untreated explants (relative Gli activity ± s.e.m.). Between 0.1 nM and 1 nM Shh the level of Gli activity at 6 h is a function of Shh concentration. (C) Temporal dynamics of Gli activity in explants exposed to Shh. The fold increase in Gli activity (relative Gli activity ± s.e.m.) measured with GBS-Luc in [i] explants treated with either 0.25 nM Shh (blue) or 2 nM Shh (green) for the indicated times. Data for 1 nM Shh (red dashed line) taken from Dessaud et al. (2007) [24] is included for comparison. Cells become adapted to these concentrations of Shh; moreover the time taken for Gli activity to return to less than 4-fold above pre-stimulus levels (inset) when exposed to the indicated concentrations of Shh is proportional to Shh concentration.

Intracellular signal transduction by Shh results in increased transcriptional activity of the Gli family of zinc finger transcription factors [19]. Our previous studies demonstrated that exposure of neural cells to concentrations of Shh in excess of 1 nM, which induce p3 and pMN progenitors, saturate intracellular signalling and generate similar high levels of Gli activity during the first 12 h [24]. Lower concentrations of Shh are associated with the production of several subtypes of interneurons, dorsal to MNs, including V0, V1, and V2 neurons [34]. We therefore assayed the profile of Gli activity induced by lower concentrations of Shh. For this, intermediate neural plate ([i]) explants from Hamburger and Hamilton (HH) stage 10 chick embryos in ovo electroporated with a Gli reporter (GBS-luc; Figure 1A; see Materials and Methods; [24]) and normalization plasmids were cultured in the presence of 0.1–2 nM Shh. The level of Gli activity in these explants was measured 6 h after the start of culture and compared to the levels of Gli activity in explants cultured in the absence of Shh (Figure 1B). These data indicated that saturating levels of signal transduction, corresponding to Gli activity ∼25-fold higher than basal levels, were reached at ∼1 nM Shh (Figure 1B). This is consistent with our previous findings [24]. At concentrations less than 1 nM Shh, however, the level of Gli activity was a function of Shh concentration and half maximal levels of Gli activity (10–15-fold induction) were obtained between 0.25 nM and 0.5 nM Shh (Figure 1B).

We next compared the dynamics of Gli activity in explants exposed to 0.25 nM and 2 nM Shh for 6–24 h (Figure 1C). For both concentrations, maximum levels of Gli activity were recorded at ∼6 h and subsequently decreased over time. At 2 nM Shh, Gli activity level at 6–12 h was similar to that generated by 1 nM Shh. In contrast to 1 nM Shh, however, cells exposed to 2 nM Shh contained higher levels of Gli activity at 18–24 h, indicating that the rate of signalling decline is inversely correlated with morphogen concentration. This is consistent with the “temporal adaptation” mechanism [24]. At 0.25 nM, Shh signalling pathway was not saturated. Thus levels of Gli activity reached in this condition were lower; nevertheless the level of signalling declined over time and was indistinguishable from pre-stimulus levels by 18–24 h. Consequently, lower concentrations of Shh sustain signal transduction above basal levels for shorter periods of time (Figure 1C, inset). Together these data suggest that exposure of neural cells to Shh generates distinctive temporal profiles of intracellular signalling. Gli activity reaches a peak within ∼6 h of exposure to Shh, and these peak levels correspond linearly with Shh concentration for low concentrations and are saturated at high concentrations. Subsequently, for all concentrations, intracellular signalling declines from the peak as cells adapt and the length of time it takes for Gli activity to return to pre-stimulus levels increases with Shh concentration.

### The Duration of Shh Signalling Influences V0–V2 Neuron Generation

**Figure 2:**
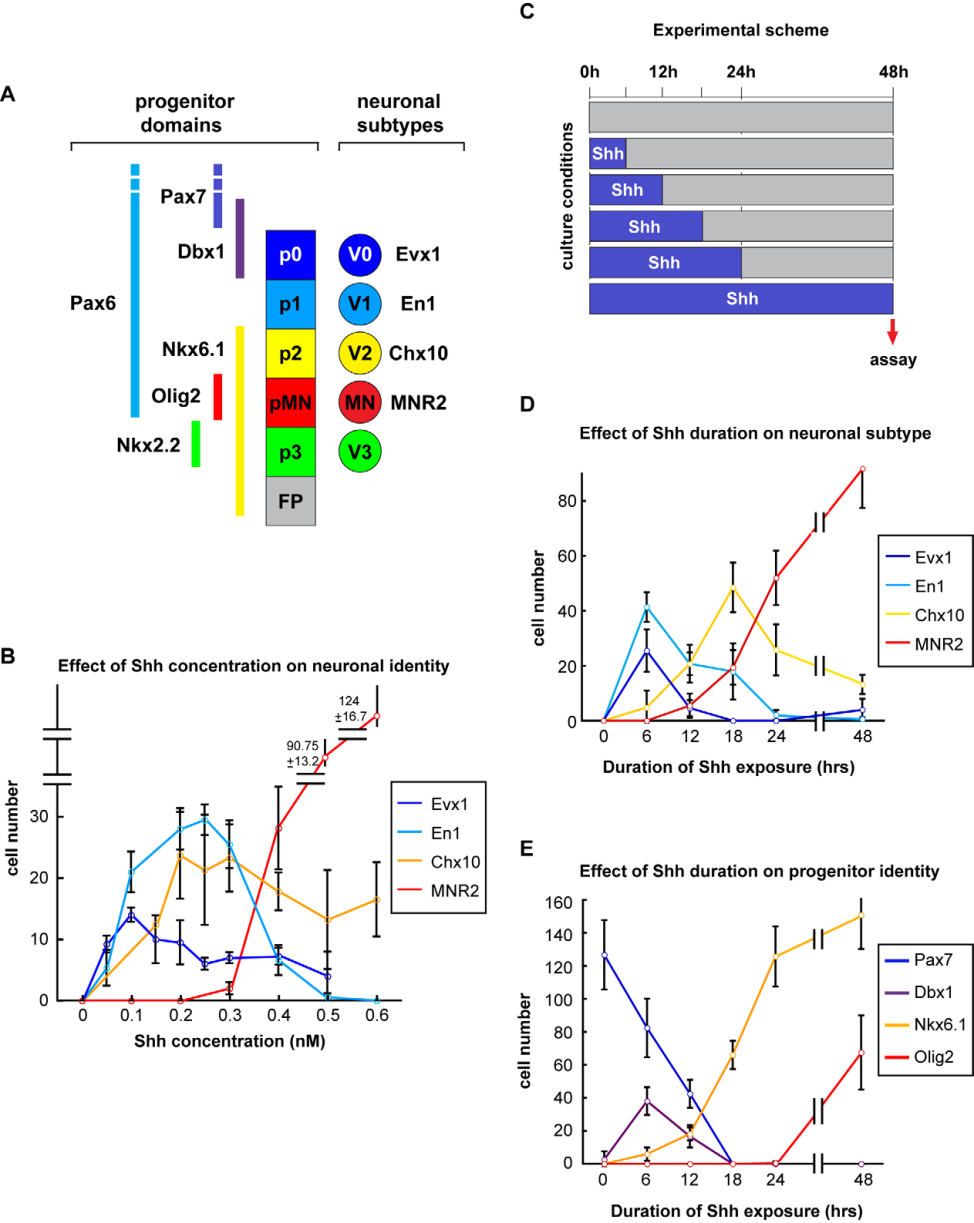
Effects of Shh concentration and duration of Shh exposure on the assignment of the ventral progenitor and neuronal identities. (A) Schematic of expression patterns of progenitor and neuronal subtype marker proteins in the ventral neural tube. FP, floor plate. (B) Quantification of cells expressing Evx1, En1, Chx10, and MNR2, markers of V0, V1, V2, and MNs, respectively, in [i] explants cultured for 48 h in the indicated concentrations of Shh (n≥4; cells/unit ± s.d.). (C) Summary of experimental scheme for (D) and (E). Explants were exposed to 0.5 nM Shh for 6–48 h (blue columns), then transferred to fresh media lacking Shh (gray columns) and incubation continued until a total of 48 h had elapsed. (D) Quantification of cells expressing Evx1, En1, Chx10, and MNR2 after 48 h of ex vivo culture of [i] explants exposed to 0.5 nM Shh for the indicated times (n≥4; cells/unit ± s.d.). (E) Quantification of cells expressing the progenitor markers Pax7, Dbx1, Nkx6.1, and Olig2 after 48 h of ex vivo culture of [i] explants exposed to 0.5 nM Shh for the indicated times (n≥4; cells/unit ± s.d.).

We next assayed how the observed dynamics of Shh signalling regulate the specification of different neuronal subtypes. We focused our attention on Shh concentrations <1 nM and the generation of V0, V1, and V2 neurons within the intermediate region of the neural tube (Figure 2A) [34]. Explants were exposed to 0.05–0.5 nM Shh for 48 h, then assayed for Evx1, En1, Chx10, and MNR2 expression, markers of V0, V1, and V2 neurons and MNs, respectively (Figure 2B, S1A, S1B, S1C, S1D, S1E, S1F; [35],[36],[37],[38]). In the absence of Shh, most cells express Pax7 (Figure S2; [39]) and few, if any, cells expressed V0–V2 or MN markers (Figure 2B). Addition of 0.05–0.3 nM Shh resulted in a reduction in Pax7 expression, induction of the intermediate progenitor marker Dbx1 (Figure S2), and the production of cells expressing Evx1, En1, or Chx10 (Figure 2B). There was a trend for the induction of increasing numbers of more ventral cell types as Shh concentration was increased. For instance, the production of V0 neurons peaked at ∼0.1 nM while the maximum numbers of V1 neurons were observed at ∼0.25 nM Shh and MNs were only induced in significant numbers by concentrations of Shh >0.4 nM Shh (Figure 2B). Nevertheless, a mixture of neuronal subtypes was generated in response to all Shh concentrations tested in the range of 0.05–0.4 nM. Thus, these data indicate that, although low concentrations promote the generation of V0–V2 neurons, incremental differences in concentration are not sufficient to generate distinct neuronal subtypes. Since the Shh concentrations used in these experiments produced distinct levels of Gli activity (Figure 1B), the data suggest that the level of signalling is not sufficient to separate the specification of V0, V1, and V2 neurons.

As different Shh concentrations could not delineate clear transitions in the production of different neuronal subtypes, we asked whether the length of time cells are exposed to Shh could distinguish the generation of ventral neurons. For this, explants were exposed to 0.5 nM Shh for fixed times ranging from 6 h to 48 h. After the indicated period of Shh exposure, Shh was removed and replaced with media lacking Shh; neuronal subtype identity was then assayed in all explants at 48 h (Figure 2C, 2D). Exposure to Shh for only the first 6 h of the 48 h culture period was sufficient to induce V0 and V1 neurons, but few, if any, V2 neurons were generated (Figure 2D). Increasing the duration of Shh signalling resulted in the progressive generation of more ventral cell types: 12 h exposure resulted in V2 generation and a marked reduction in V0 generation, while 18 h exposure was required for the appearance of MNs (Figure 2D). Thus different durations of signalling influenced the neuronal subtype generated in response to a fixed Shh concentration such that more ventral neuronal subtypes were generated in explants exposed for longer periods of time. Notably, different durations of Shh signalling generated well-separated peaks of V0 and V2 neurons, in contrast to the overlapping peaks of neuron production following exposure to different Shh concentrations (compare Figure 2B with 2D). Nevertheless there was still significant overlap in the generation of V0 and V1 neurons (Figure 2D). This might reflect limitations in the resolution of the explant assay or the action of additional signals in the generation of these cell types [40].

To confirm the involvement of signal duration in the assignment of positional identity, we assayed the expression of transcription factor markers of p0–p2 progenitors, which generate V0–V2 neurons. Explants were assayed at 48 h after exposure to 0.5 nM Shh for different periods of time (Figures 2C, 2E, S1G, S1H, S1I, S1J). V0 neurons are generated from progenitors that express Dbx1 ventral to the Pax7 boundary; progenitors of V1 neurons express Pax6 but lack Nkx6.1 and Dbx1 expression; V2 progenitors express Nkx6.1 but not Olig2 (Figure 2A; [41]). Consistent with the profile of neuronal subtype generation, increasing the duration of Shh signalling resulted in a gradual ventralization of progenitor cells. In the absence of Shh, progenitor cells expressed Pax7. Exposure to Shh for 6 h induced Dbx1 expression and decreased the number of Pax7 expressing cells by ∼40% (Figure 2E). Longer periods of exposure resulted in a gradual decrease in Dbx1 and Pax7 expression and an increase in the expression of Nkx6.1; finally Olig2 expression, the pMN marker, was detected (Figure 2E). Thus, similar to neuronal subtype identity, the response of the transcriptional markers of progenitor domains to different durations of Shh exposure corresponds to their DV position in the neural tube.

We sought to rule out the possibility that the positional identities induced by different times of Shh exposure were the result of temporal changes in the competence of progenitors. If this were the case, the time at which cells received Shh signalling, rather than duration for which they were exposed to Shh, would determine positional identity. We therefore assessed the effect of adding Shh to explants at different time points. Explants were cultured in the absence of Shh for 12–24 h and then exposed to 0.5 nM or 4 nM Shh for an additional 6–24 h (Figure S3). Assaying the expression of Dbx1, Nkx6.1, Olig2, and Nkx2.2 revealed that the response of explants was offset by the same amount of time that the addition of Shh was delayed. For example, Dbx1 and Nkx6.1 were induced by exposure to 0.5 nM Shh for 6 h and 18 h, respectively, regardless of whether Shh exposure was initiated at 0 h or 24 h after the start of the culture period (Figure S3A). Similarly, 12 h of exposure to 4 nM Shh induced Olig2, while longer times were required for Nkx2.2 induction, whether Shh was added immediately after explanting or following 12–24 h ex vivo (Figure S3B). These data argue against an intrinsic timing mechanism that over time changes the competence of progenitors to generate different neuronal subtypes. Instead the data provide strong support for the idea that the duration of Shh signalling plays a central role in determining positional identity. Thus, throughout the ventral neural tube, progressively more ventral fates are generated as the concentration and time of exposure to Shh are increased.

### Progressive Assignment of Progenitor Identity in the Ventral Neural Tube

**Figure 3:**
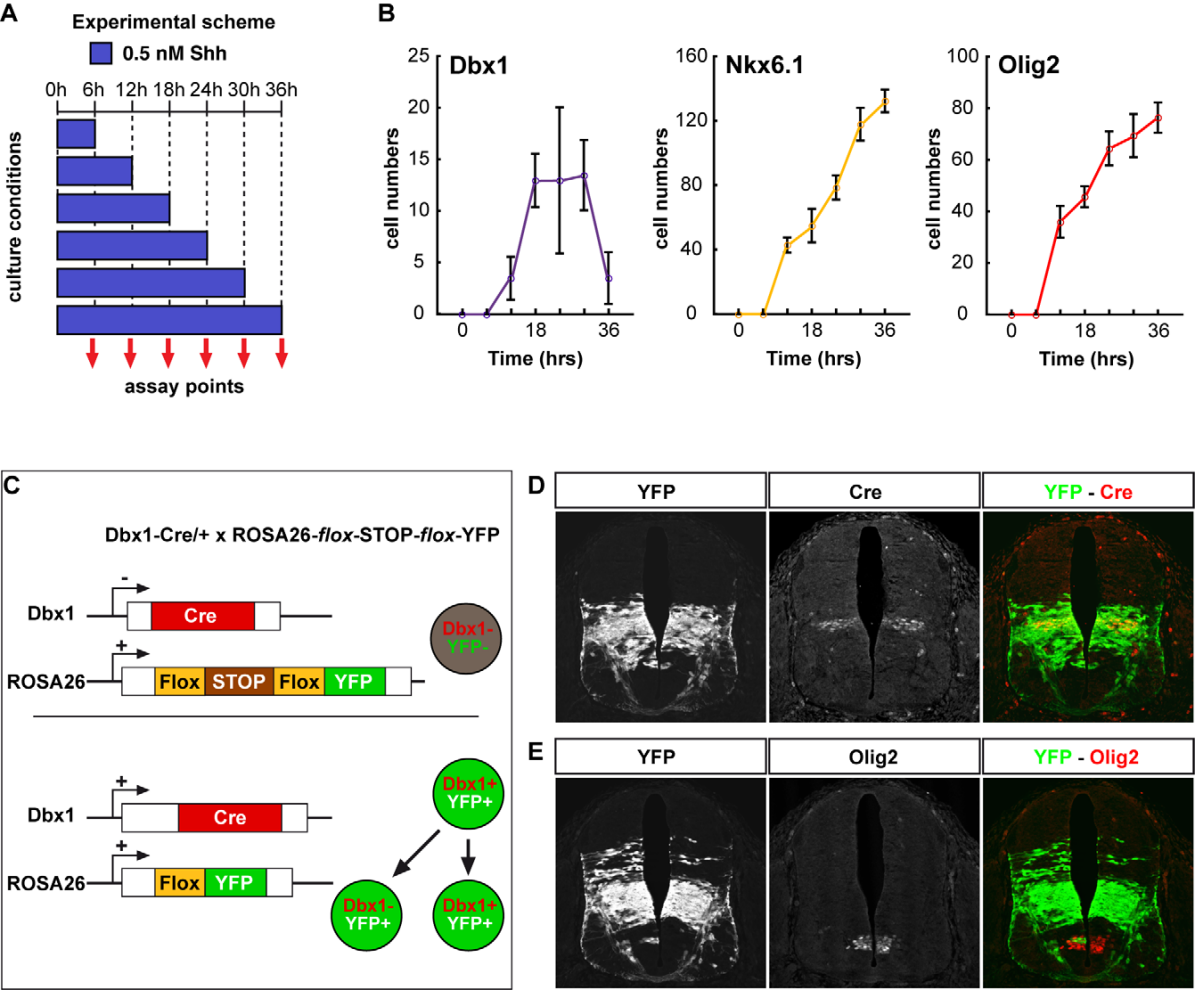
Progressive induction and refinement of ventral progenitor identity in vitro and in vivo. (A) Experimental scheme. Explants were exposed to 0.5 nM Shh for 6–36 h (blue columns), then fixed directly and assayed for the expression of Dbx1, Nkx6.1, and Olig2. (B) Quantification of cells expressing Dbx1, Nkx6.1, and Olig2 in [i] explants exposed to 0.5 nM Shh for the indicated time (n≥4; cells/unit ± s.d.). Dbx1 expression peaked by 18 h, while the number of cells expressing Nkx6.1 and Olig2 continued to increase over time. (C) Strategy for permanently marking cells that have expressed Dbx1. Mice harbouring a Cre recombinase in the Dbx1 locus were crossed with ROSA26^flox^STOP^flox^YFP mice. In cells that express Dbx1, expression of Cre results in a recombination event removing the STOP cassette from ROSA26^flox^STOP^flox^YFP, thus allowing LacZ expression. The ubiquitously expressed ROSA26 locus directs continued YFP expression even after Dbx1 is downregulated. (D, E) Two examples of e11.5 embryos containing Dbx1cre and ROSA26^flox^STOP^flox^YFP assayed for YFP and either cre (D) or Olig2 (E) expression. The expression of cre from the Dbx1 locus is limited to the p0 progenitor domain in the intermediate region of the neural tube. YFP expression is observed within the cre expressing progenitors but also in more ventrally located progenitors including some that express the pMN marker Olig2. This indicates that Dbx1 is transiently expressed in progenitors ventral to later expression pattern.

To test directly whether progenitors progressively adopt more ventral identities as the duration of Shh exposure is increased, we assayed the dynamics of Dbx1, Nkx6.1, and Olig2 expression in [i] explants exposed to 0.5 nM Shh for 6–36 h (Figure 3A). At 18 h, explants expressed the three markers (Figure 3B). Exposure to 0.5 nM Shh for longer periods of time resulted in the gradual increase in the number of Olig2 and Nkx6.1 expressing cells and a reduction in Dbx1 expression and only a small number of V0–V2 neurons were present in explants exposed for 36 h to 0.5 nM Shh (Figures 3B, S4A, S4B, S4C). These data suggest that continued Shh signalling after 18 h promotes the acquisition of more ventral positional identities at the expense of intermediate fates. Notably, by 18 h the level of Shh signalling in cells exposed to 0.5 nM Shh was significantly reduced from the peaks attained at 6–12 h (Figure 1C; [24]). Nevertheless, this level of signalling appears sufficient for the progression of progenitor identity from intermediate to pMN.

A consequence of the sequential establishment of positional identity is that the ventral limit of expression of a progenitor marker such as Dbx1, which identifies p0 progenitors, should be displaced dorsally as development proceeds. In this view, cells that have expressed Dbx1 will contribute to progenitor domains ventral to p0. To test this, we took advantage of a Dbx1-Cre mouse line [42] and the inducible reporter allele ROSA26^flox^STOP^flox^YFP (Figure 3C; Materials and Methods; [43]). In these embryos the progeny of any cell that has expressed Dbx1 is indelibly marked by the expression of YFP. In embryos assayed at E11.5, the ventral limit of YFP expression was more ventral than the extant p0 domain, identified by Dbx1 (unpublished data) and Cre recombinase expression (Figure 3D), and some Dbx1 progeny were detectable within Olig2 positive progenitors (Figure 3E). Thus, Dbx1 is initially expressed in more ventral progenitors and its expression is refined, eventually becoming restricted to the p0 domain. These data provide evidence that ventral neural tube patterning proceeds via the progressive assignment of ventral identities.

### Progenitors Revert to a More Dorsal Identity when Shh Signalling Is Blocked

**Figure 4:**
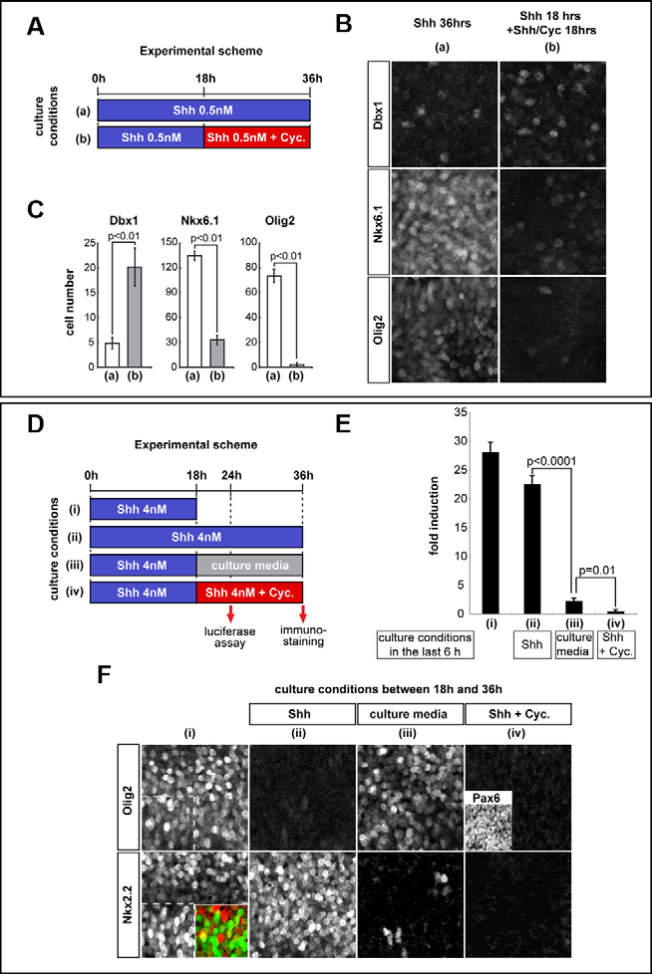
Ventral progenitors revert to a more dorsal identity when Shh signalling is interrupted. (A) Scheme for experiments in (B) and (C). Explants were exposed to 0.5 nM Shh (blue columns) for 36 h, either continuously (a) or after 18 h some were transferred to media containing 500 nM cyclopamine (Cyc.) and 0.5 nM Shh (b; red columns) to block Shh signalling. All explants were assayed 36 h after the start of culture. (B) Representative images of explants assayed for Dbx1, Nkx6.1, and Olig2 expression in each condition. (C) Quantification of cells expressing Dbx1, Nkx6.1, and Olig2 in [i] explants exposed to 0.5 nM Shh for 36 h (a) or 0.5 nM Shh for 18 h followed by 500 nM cyclopamine and 0.5 nM Shh (b) for an additional 18 h (n≥4; cells/unit ± s.d.). (D) Scheme of the experiments in (E) and (F). Explants were exposed to 4 nM Shh (blue columns) for 18 h or 36 h. After 18 h, some explants were transferred to media containing 500 nM cyclopamine and 4 nM Shh (red columns); others were transferred to basal media lacking added factors (gray columns). Explants were assayed for marker gene expression at 18 h (i) or 36 h (ii–iv) and for Gli activity at 24 h after the start of culture. (E) Fold increase in Gli activity, compared to untreated explants (relative Gli activity ± s.e.m.) measured with GBS-Luc, in [i] explants treated as indicated in the schema. At 18 h (i) and 24 h (ii) 4 nM Shh induced ∼20–25-fold increase in Gli activity. Within 6 h of blockade of Shh signalling with cyclopamine, Gli activity levels returned to pre-stimulus levels (iv). Removal of Shh resulted in a reduction of Shh signalling at 6 h (iii), however levels 3–4-fold above basal were still present at this time point. (F) Explants, cultured in the conditions indicated in (D), assayed for Olig2 and Nkx2.2 expression. After 18 h exposure to 4 nM Shh (i) explants expressed a mixture of Olig2 and Nkx2.2 with some cells co-expressing both markers (inset). Treatment with 4 nM Shh for 36 h resulted in the repression of Olig2 and the exclusive expression of Nkx2.2 (ii). In explants assayed at 36 h in which 4 nM Shh had been removed 18 h earlier, Nkx2.2 expression had largely been lost while Olig2 expression was maintained (iii). In contrast, blockade of Shh signalling with cyclopamine at 18 h resulted in the loss of both Olig2 and Nkx2.2 (iv); in this condition progenitors expressed high levels of Pax6.

The progressive assignment of positional identity by Shh signalling prompted us to question whether continued signalling is required to maintain specific ventral identities once they are induced. To test this, we blocked Shh signalling in explants at different time points using the inhibitor cyclopamine [32],[44], an antagonist of Smoothened, the essential intracellular transducer of the pathway [45],[46]. After explants had been exposed to 0.5 nM Shh for 18 h, they were cultured for an additional 6 h, 12 h, or 18 h in fresh media containing 500 nM cyclopamine (Figures 4A–4C, S3D). Explants that had been exposed to 0.5 nM Shh for 18 h contained significant numbers of Dbx1, Nkx6.1, and Olig2 expressing cells (Figure 3B). However, when Shh signalling was inhibited from 18 h, a marked reduction in the expression of ventral progenitor markers Nkx6.1 and Olig2 was apparent at 36 h (Figure 4B, 4C), compared to explants exposed to 0.5 nM Shh for 18 h (Figure 3B) or for 36 h (Figure 4B, 4C). Consistent with this, MN production was reduced when signalling was blocked at 18 h (Figure S4B, S4C). Notably, Olig2 expression was more severely affected than Nkx6.1 (Figure 4C). This suggests that Olig2 is more sensitive than Nkx6.1 to removal of signalling and that the remaining Nkx6.1 expressing cells in these explants represent p2 progenitors. Concomitant with the decrease in ventral progenitor markers, the numbers of Dbx1 expressing progenitors and V0–V2 neurons increased (Figure 4C, S4B, S4C). This change in progenitor identity inversely correlated with the concentration of Shh required for induction and indicates that cells gradually revert to a more dorsal identity when Shh signalling is blocked. These data suggest, therefore, that Shh signalling is required not only to induce ventral identity but also to maintain the identity after it has been assigned.

We asked whether the requirement for Shh signalling to maintain progenitor identity is a general property of the response of neural progenitors and is therefore also observed in cells exposed to higher Shh concentrations. For these experiments, explants were exposed to 4 nM Shh for 18 h, and then placed into fresh media containing either 4 nM Shh, or lacking Shh, or containing 4 nM Shh and 500 nM cyclopamine, to block signalling. Incubation of explants was continued for either an additional 6 h, at which time point the level of Gli activity assayed, or an additional 18 h in order to assay Olig2, Pax6, and Nkx2.2 expression (Figure 4D). Six hours after the addition of 500 nM cyclopamine, Gli activity levels had returned to basal levels (Figure 4E, iv). By contrast, 6 h after removal of Shh from the medium, the levels of Gli activity were significantly lower but remained 2–3-fold above basal levels (Figure 4E, iii). After 18 h exposure to 4 nM Shh, explants expressed a mixture of Olig2 and Nkx2.2 (Figure 4F, i; see also [24]); a few cells expressed both Nkx2.2 and Olig2 but the majority of cells expressed one or the other marker. In explants in which exposure to 4 nM Shh was maintained for 36 h, Nkx2.2 expression was consolidated and Olig2 was downregulated (Figure 4F, ii). By contrast, in explants in which Shh was removed and incubation continued for an additional 18 h in the absence of Shh, most cells reverted to expressing Olig2 alone and Nkx2.2 had largely been downregulated (Figure 4F, iii). This indicates that Gli activity levels 2–3-fold above basal are sufficient to maintain Olig2, but not Nkx2.2 expression. The complete inhibition of Shh signalling with 500 nM cyclopamine resulted in the downregulation of both Nkx2.2 and Olig2 (Figure 4F, iv). These explants expressed high levels of Pax6, a marker of the intermediate neural tube (Figure 4F, iv; [25]). These data demonstrate that continuous Shh signalling is required for maintaining the identity of all ventral neural progenitors. In addition, it suggests that the maintenance of different target genes requires different thresholds of Gli activity.

These data provide an explanation for the discrepancy in the identity of progenitor and post-mitotic cells observed in the data from initial experiments using [i] explants (Figure 2D, 2E). In these experiments, the subtype identity of neurons generated by a specific duration of signalling did not always correspond to the progenitor markers expressed in the same conditions. For example, 18–24 h exposure to Shh was sufficient to induce large numbers of MNs in explants assayed at 48 h, however there was no detectable Olig2 expression in explants assayed in the same conditions. This is consistent with the idea that pMN progenitors were present in these explants at an earlier time point, but by the time the assay was performed, these progenitors had disappeared, leaving behind the apparently anomalous post-mitotic neurons they had generated. Together, these data identify a function for Shh signalling in maintaining progenitor identity and suggests a dual role for Shh signalling in the neural tube: first to assign progenitor identity and then to maintain the established positional identity of ventral progenitor cells.

### Prolonged Exposure to Shh Signalling Is Required In Vivo for Ventral Patterning

**Figure 5:**
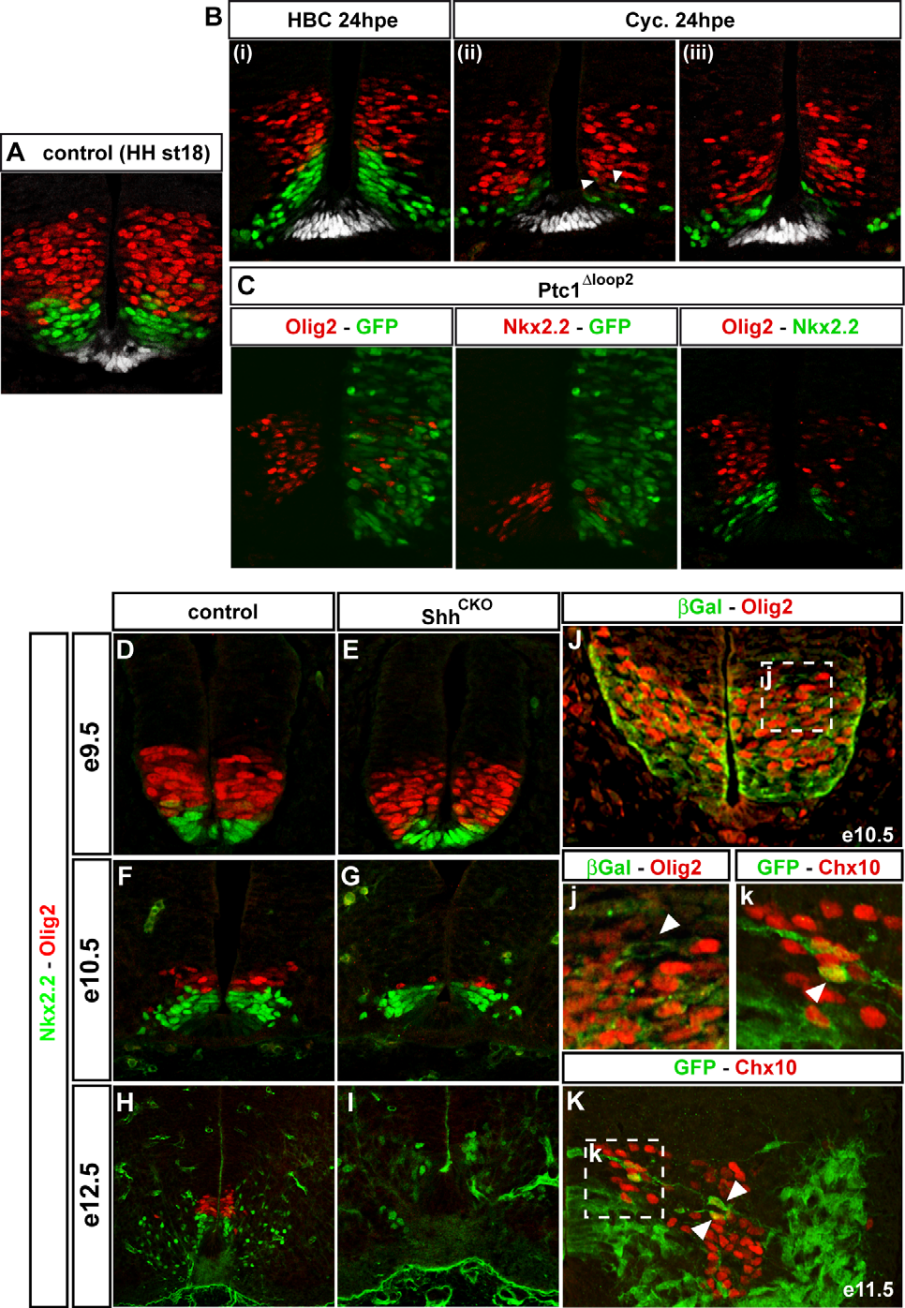
Shh signalling is required to maintain ventral progenitor pattern in vivo. (A) Expression of Olig2 (red), Nkx2.2 (green), and Arx (which marks specifically floor plate cells; white) [84] in HH st.18 chick embryos. (B) In ovo blockade of Shh signalling using cyclopamine (Cyc). HH st.18 embryos were treated with vehicle (HBC; i) or cyclopamine (ii, iii) in ovo and incubated for 24 h. Assay for Nkx2.2 (green), Olig2 (red), and Arx (white) expression at anterior thoracic levels revealed a ventral shift and marked decrease in the number of cells expressing Nkx2.2 and Olig2 compared to HBC control embryos and HH st.18 embryos. In addition, some Olig2 and Nkx2.2 double-positive cells can be seen, close to the floor plate, in the Nkx2.2-positive domain (arrowheads) in embryos exposed to cyclopamine. (C) In ovo inhibition of Shh signalling by expression of Ptc1^Δloop2^. HH st.18 embryos in ovo electroporated with Ptc1^Δloop2^IRES-GFP were assayed 24 h after transfection for the expression of Olig2 and Nkx2.2 and GFP, which marks transfected cells. Cells expressing Ptc1^Δloop2^ downregulated Olig2 and Nkx2.2 expression in a cell autonomous manner. (D–I) Expression of Olig2 (red) and Nkx2.2 (green) at the brachial levels in the mouse embryos in which the conditional allele of Shh^flox/flox^ has been deleted with Brn4cre (conditional knockout of Shh; Shh^CKO^). At e9.5, expression of both Olig2 and Nkx2.2 in Shh^CKO^ embryos (E) is indistinguishable from that in wild type (D). At e10.5, however, there was a marked reduction in the mutants (G), compared to the control littermates (F), and at e12.5, the expression had completely declined (H, I). (J) Lineage tracing of Olig2 progeny. E10.5 embryos containing Olig2-Cre and ROSA26^flox^STOP^flox^LacZ assayed for β-Gal and Olig2 expression. β-Gal expression is observed in a small number of progenitors dorsal to the pMN domain that do not express Olig2 (inset j). This suggests that Olig2 is transiently expressed dorsal to its normal domain of expression. (K) Assaying Olig2-Cre; ROSA26^flox^STOP^flox^GFP embryos at e11.5 revealed some GFP cells co-expressing the V2 marker Chx10 (arrowheads; inset k). This is consistent with the transient expression of Olig2 in progenitor cells of the p2 domain that then generate V2 neurons.

The ex vivo data prompted us to ask whether an extended period of Shh signalling is required in vivo to maintain appropriate patterns of gene expression. We first used cyclopamine to block Shh signalling by administering the drug in ovo to HH st.18 embryos. At this developmental stage, corresponding to ∼36 h after the time at which intermediate regions are explanted from embryos, progenitor domains are well established (Figure 5A) and neurogenesis is under way. Expression of progenitor markers of the ventral neural tube was then analyzed 24 h later, after administration of cyclopamine or vehicle alone; this corresponded to ∼HH st.22. At thoracic levels, altered ventral patterning was observed in 80% of the embryos exposed to cyclopamine (Figure 5B). In these embryos, the number of Nkx2.2+ cells was markedly reduced and the domain of Olig2 shifted ventrally (Figure 5B, ii). Furthermore, the pMN domain was affected in 60% of the samples, with fewer cells expressing Olig2 (*n* = 12; Figure 5B, iii). Thus, similar to the ex vivo results, continued Shh signalling appears to be required to maintain the appropriate pattern of progenitor identities in the ventral neural tube, and if Shh signalling is interrupted cells transform into a more dorsal identity.

To corroborate these data, we blocked Shh signalling, in ovo, by electroporation of Ptc1^Δloop2^, a dominant active version of the Shh receptor that cell autonomously blocks intracellular Shh signal transduction (Figure 5C; [47]). Embryos transfected with Ptc1^Δloop2^ at HH st.18 were assayed for the expression of progenitor markers of the ventral neural tube 24 h later, at ∼HH st.22. Similar to the results of cyclopamine exposure, the inhibition of Shh signalling with Ptc1^Δloop2^ resulted in the inhibition of Nkx2.2 and Olig2 expression (Figure 5C). The effects of Ptc1^Δloop2^ appeared cell autonomous, as only transfected cells displayed obvious changes in gene expression, consistent with the cell autonomous inhibition of Shh signalling imposed by Ptc1^Δloop2^ [47].

To test whether an extended period of Shh signalling is also necessary in the mouse neural tube, we deleted Shh from the floor plate at ∼e9.5. Mice containing a conditional null allele for Shh (Shh^flox/flox^; [48]) and a Brn4cre transgene (*Bcre32* [Tg(Pou3f4-cre)32Cre]; [49]), which directs cre expression to neural progenitors from ∼e9.0, were used. We confirmed that Shh is lost from the cervical neural tube of Shh^flox/flox^;Brn4cre embryos by e10.5 but left notochord expression of Shh unaffected (Figure S5A, S5B). Moreover, in contrast to embryos lacking Gli2 [50], in which the notochord remains abutting the neural tube, in Shh^flox/flox^;Brn4cre embryos the notochord separated normally from the overlaying neural tissue. Assaying expression of Nkx2.2 and Olig2 at e9.5 revealed that the induction and extent of expression of both proteins were similar in Brn4cre;Shh^flox/flox^ embryos and littermate controls (Figure 5D, 5E). By contrast, analysis of e10.5 Brn4cre;Shh^flox/flox^ embryos revealed a marked decrease in the number of cells expressing Nkx2.2 and Olig2 and a ventral retraction in the domains of expression (Figure 5F, 5G). Notably, the expression of Olig2 expression was more affected than Nkx2.2. In addition the number of cells expressing Nkx6.1 was decreased; concomitantly there was a ventral shift in the expression of Dbx1 and Pax6 (Figure S5C, S5D; unpublished data). By e12.5 embryos, the expression of both Nkx2.2 and Olig2 was severely affected in Brn4cre;Shh^flox/flox^ and only a few cells expressing either marker could be detected (Figure 5H, 5I). Thus, similar to chick, prolonged exposure to Shh is required to maintain normal ventral pattern in the neural tube. Moreover, these data identify a crucial role for floor plate derived Shh in the patterning of the neural tube. Together, these results indicate that, even after progenitor identity has been assigned, continued Shh signalling is necessary to maintain progenitor identity in vivo in the ventral neural tube.

### Progenitor Identity Reversion Occurs During Normal Neural Tube Development

The continuous requirement for Shh signalling in vivo, coupled with the plasticity of progenitor identity in explants, raised the possibility that during normal embryonic development some progenitor cells may transiently express a transcriptional code characteristic of a more ventral progenitor population before reverting to a more dorsal identity. To test this, we used Olig2Cre mice to mark the progeny of cells that have expressed Olig2 [24]. Examining E10.5 mice embryos harbouring the Olig2cre allele and the ROSA26-flox-STOP-flox-lacZ or the ROSA26-flox-STOP-flox-GFP lineage tracers [51] revealed cells expressing the lineage marker within the pMN domain and their MN progeny (Figure 5J, 5K). In addition, however, a small number of cells positive for the reporter were observed dorsal to the pMN domain. These were located adjacent to the dorsal limit of the pMN domain, defined by Olig2 expression, but did not express Olig2 protein (Figure 5J, j). This suggests that some cells residing in the p2 progenitor domain transiently expressed Olig2 and activated Cre expression. This was confirmed by examining e11.5 embryos. In these embryos a few Chx10 and GATA3 expressing V2 neurons, which are generated from Olig2 negative, p2 progenitors, contained the reporter, indicating that they had been generated from progenitors that had previously expressed Olig2 (Figure 5K, k and unpublished data). Together these data confirm the plasticity of progenitor identity in vivo and emphasize the dynamic way in which positional information is supplied in the neural tube.

## Discussion

**Figure 6:**
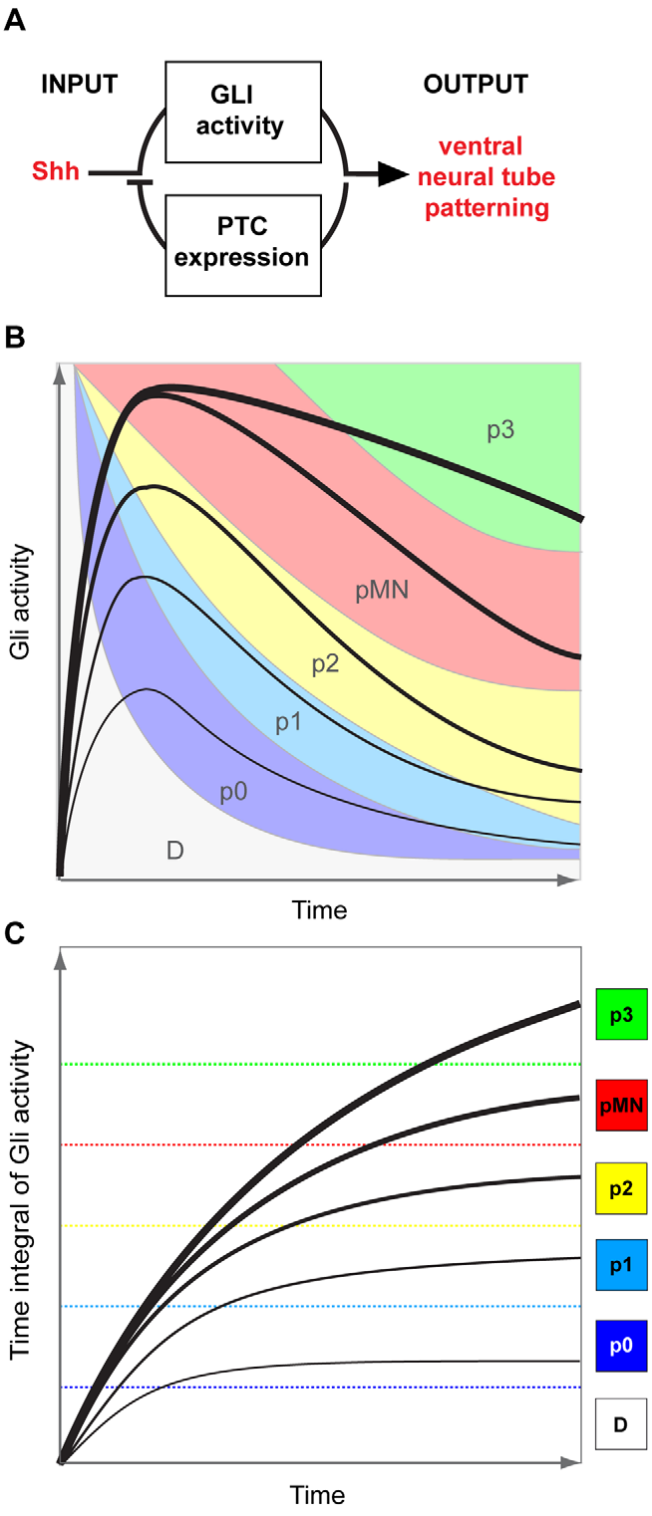
Working model for the provision of positional identity by Shh signalling. (A) A Shh induced negative feedback loop involving Gli mediated transcriptional induction of the Shh receptor Ptc1 produces a temporally dynamic output of Gli activity in response to fixed Shh input. (B) A state space model of ventral neural tube patterning. The range of time (*x*-axis) and levels (*y*-axis) of Gli activity necessary to induce each positional identity (p0–p3 and pMN) are illustrated by the coloured regions on the graph. This indicates that more ventral progenitor identities require higher levels and longer durations of Gli activity. On to this state space representation, the profile of Gli activity generated by different concentrations of Shh is indicated by the black lines. The incrementally thicker lines represent the profile generated by increasingly higher concentrations of Shh. The diagram shows how the differing profiles of Gli activity generate different positional identities in a progressive manner in the neural tube. (C) Positional information corresponds to the time integral of Gli activity. The time integral of Gli activity, equivalent to the area under the curves in (B), provides the correlate of positional information. Increasingly more ventral progenitors are generated at correspondingly higher time integrals of Gli activity.

In this study we provide evidence that the interpretation of Shh morphogen gradient in the neural tube is dynamic and entails a prolonged period of intracellular signalling to elaborate and maintain gene expression in progenitor cells. Shh induced intracellular Gli activity is required for the patterning of progenitors [22],[52]. We show that the Gli activity in neural cells reaches a peak ∼6 h after exposure to Shh. Below 1 nM Shh, the intensity of this peak of Gli activity correlates with the concentration of Shh; above 1 nM Shh intracellular signalling appears saturated (Figure 1B). For all Shh concentrations, however, cells progressively adapt their response to Shh, resulting in a gradual decline in Gli activity levels from 6 h onwards. Thus, the time it takes for Gli activity to return to basal levels (i.e. “the duration of signalling”) is proportional to Shh concentration for all concentrations. Hence, different concentrations of Shh generate distinct temporal profiles of Gli activity (Figure 1C). The features of this profile—the duration and the level of Gli activity—are used to allocate positional identity to neural progenitors (Figure 6). Progressively higher levels and longer durations of intracellular Gli activity specify more ventral identities (Figures 2, 3) and, even for the interneurons that are generated by below saturating concentrations of Shh, the duration of signalling plays a key role. These data indicate, therefore, that the temporal adaptation mechanism of morphogen interpretation allocates positional identity throughout the ventral neural tube. This offers a unifying view of ventral neural tube patterning that differs significantly from conventional models of morphogen action [3],[24]. Consistent with the dynamic aspect of this model, Shh target genes initially occupy territories that are ventral to their final positions and are progressively refined dorsally over time (Figure 3D, 3E). Moreover, even after a positional identity has been assigned to a cell, it remains susceptible to change. For the more ventral progenitor domains, Gli activity above basal levels is required to maintain an identity (Figures 4, 5). Thus, blockade of signalling results in ventral progenitors losing their identity and acquiring an antecedent, more dorsal identity. This emphasizes a second important difference between the mechanisms of Shh interpretation and standard morphogen models, which assert that positional identity of cells can ascend but not descend a gradient [3],[53]. Together, the data suggest a model in which the positional information provided by Shh can be represented by the time integral—the cumulative level and duration—of signalling and the identity of progenitors is assigned in a progressive and dynamic manner.

The use of signal duration in the assignment of positional identity contrasts with other models of morphogen action that contend that the level of intracellular signalling is the main, or sole, factor that determines the response [3]. For example, different levels of nuclear Smad activity, the transcriptional mediator of TGFβ signalling, have been proposed to be sufficient to mediate the graded response necessary for mesoderm patterning [54],[55],[56]. Similarly, the graded response of cells to Dpp in Drosophila appears to correlate with the amount of activated Mad transcription factor [57],[58],[59] and the anterior-posterior patterning of Drosophila embryos depends on the amount of Bcd protein in each nucleus [60],[61]. In the case of Shh signalling, the use of duration as well as level of signalling to provide positional information introduces an additional dimension to the process that could be relevant to other responding tissues. Notably, anterior-posterior patterning of the developing limb appears to depend on both a concentration and temporal gradient of Shh signalling [62],[63],[64] and, analogous to the neural tube, the specification of digit identity is progressive with the sequential induction of more posterior identities [65]. Likewise, Hh signalling in the wing disc of Drosophila embryos results in the induction of higher response genes later than lower response genes [66]. Thus, the mechanism of morphogen interpretation operating in the neural tube might be common to other tissues patterned by graded Hh signalling. Furthermore, since negative feedback is a general feature of most signal transduction pathways [67], the graded response of cells to other morphogens might result in similar temporal dynamics and exploit analogous mechanisms.

What determines the temporal dynamics of signalling in responding cells? The Shh dependent upregulation of Ptc1, and possibly other inhibitors of Shh signalling, results in the cell autonomous desensitization of cells to Shh over time [23],[24]. This negative feedback is likely to play a significant part in defining the signalling kinetics in responding cells. It would account for the temporal profile and in particular the rate of decline, and hence the duration of signalling, in cells exposed to a fixed concentration of Shh. However, in addition to negative feedback, the period of time a cell is exposed to Shh will also determine the duration of signalling. Progenitors in vivo are subject to a changing gradient of Shh and as a result the time that Shh is available might limit the duration of signalling [14]. This could be particularly relevant in intermediate regions of the neural tube, which generate V0–V2 neurons. It takes a longer time for the Shh gradient to extend to these positions [14] and then the growth of the neural tube and the sequestration of Shh ventrally might limit the period of time for which significant concentrations of Shh are maintained at this distance [23],[26],[47]. In agreement with this idea, significant numbers of V0 and V1 neurons are only generated in vitro when explants are exposed to Shh for less than 18 h (Figure 2D). Longer exposure results in cells switching identity towards more ventral fates. A similar model has been proposed for the temporal changes in Hh distribution in the anterior compartment of the Drosophila wing disc [68]. In this view, the duration of Shh exposure is decisive for specifying the intermediate identities, p0–p2, while the more ventral pMN and p3 identities are chiefly dependent on maintaining appropriate high concentrations of Shh, which are transformed into corresponding periods of intracellular signal transduction by negative feedback. However, the critical test of this model awaits the capability to assay directly Shh protein distribution and intracellular signalling in vivo in the developing neural tube.

The central role of Shh in providing positional information to neural progenitors does not exclude the possibility that other extrinsic signals contribute to ventral neural tube patterning. Members of the BMP and Wnt families as well as Notch and retinoic acid signalling have been implicated in aspects of neural tube development and cell fate allocation [6],[40]. It is possible that these interact, either directly or indirectly, with Shh signalling to refine and modify the positional identity provided by Shh signalling. This could increase the precision or reliability of patterning in vivo. In this context, it is notable that the in vitro generation of V0 and V1 neurons overlap in explants exposed to Shh (Figure 2D). Both retinoic acid and Notch signalling have been implicated in the generation of these neuronal subtypes and it is possible that one or both of these signals are involved in the spatially segregated generation of these interneurons in vivo [40],[69].

The dynamic nature of Shh mediated pattern formation is emphasized by the requirement for continued Shh signalling to maintain the more ventral progenitor identities even after the gene expression profiles that define these progenitor domains have been induced. Thus, progenitors of MNs and V3 neurons revert to more dorsal identities if signalling is interrupted. This provides an explanation for the requirement for Shh signalling up to the last cell division in order to specify MN subtype identity [39]. Importantly, this also identifies a role for floor plate produced Shh for DV patterning of neural progenitors despite the initial induction of ventral gene expression by Shh secreted from the notochord [14]. Moreover, reversibility of the expression of Hh target genes has also been observed in other tissues, such as the Drosophila wing disc [70], suggesting it is a common feature of the pathway. It remains to be determined whether the expression of Nkx2.2 and Olig2 eventually becomes independent of continued Shh signalling in the neural tube and if so at what time point. Nevertheless, the extended requirement for Shh signalling to maintain correct tissue pattern highlights a difference with other morphogens. In the case of mesoderm induction by Activin [53] and BMP signalling in the telencephalon [71] cells maintain the gene expression profile associated with the highest concentration of morphogen to which they have been exposed, even if the ligand exposure is limited to as little as 20 min [53]. This has been dubbed “the ratchet effect” because cells appear to respond to increases but not decreases in morphogen concentration [3],[53]. As a result, prolonged exposure to TGF-β morphogens is unnecessary for patterning [53]. One consequence of a self-sustaining memory for the highest morphogen response is that it obviates the need to preserve a stable gradient for a long period time. This might be important in tissues that develop quickly and/or undergo movements that preclude the establishment of a stable gradient. However, cells would be vulnerable to transient fluctuations in signalling and could adopt an identity inappropriate for their position in response to a brief exposure to an anomalously high level of morphogen. Thus, the requirement for an extended period of Shh signalling to maintain positional identity in the neural tube emphasizes the ongoing refinement of progenitor boundaries during development and suggests a way to buffer fluctuations in ligand concentration and enhance the precision of the response.

The delayed induction of some Shh target genes in progenitor cells suggests that the transcriptional state of cells is important for progenitor identity specification. This points to a model where the transcriptional network downstream of Shh signalling together with the temporal profile of Gli activity in a cell accounts for differential gene expression. Hence, Gli dependent induction of some genes might take place only after changes in the transcriptional state of a responding cell has been changed by an early period of Gli activity. This would explain the delayed induction of some progenitor markers and a requirement for ongoing signalling for their expression. A well-described example of this strategy is found in the Dpp response of cells of the dorsal ectoderm of Drosophila embryos [72]. In this case, Dpp induces expression of the transcription factor Zen. Then, Zen together with continued Dpp signalling activates a second gene, Race. Thus, the expression of Race takes longer than Zen and requires Dpp signalling to be sustained after Zen is induced. This strategy for morphogen dependent gene regulation has been referred to as “sequential cell context” [73] and represents an example of a feed-forward loop [74]. In the neural tube, it is well established that the transcriptional cross-repression between target genes downstream of Shh plays a key role in defining the spatial extent of progenitor domains [21]. It seems likely that these same regulatory interactions will also be involved in the dynamics of gene expression. For example, the repression of Nkx2.2 by Pax6 determines the dorsal limit of Nkx2.2 expression [25]; moreover, Nkx2.2 expression appears to shift increasingly dorsal in mouse embryos lacking Pax6 (N.B. et al., unpublished observation). Thus both the temporal and spatial pattern of Nkx2.2 appears to be influenced by Pax6. The reciprocity of many of the cross-regulatory interactions within the transcriptional network might also account for the prolonged requirement for Shh signalling to sustain progenitor identity. In this view, continued Gli activity would be required to ensure that the extant transcriptional state of a responding cell does not revert to a previous state. This strategy of morphogen interpretation suggests a dynamic mechanism in which the positional identity of a cell is determined by the combined action of the ligand gradient and the state of the transcriptional network in the responding cell itself.

More generally, the mechanism of Shh morphogen interpretation is reminiscent of “integral feedback control,” a common control strategy in systems engineering in which the time integral of the difference between the actual output and a target output is fed back into the system to correct the output [75]. In the case of Shh signalling, the Gli dependent induction and gradual accumulation of Ptc1 could be viewed as the “time integral” that corrects the sensitivity of cells to Shh; hence increasing amounts of Ptc1 result in the increasing desensitization of cells to Shh signalling (Figure 6A, 6B). The use of integral feedback control has been noted in several biological systems where it can act as a “gain” control to allow sensing over large concentration ranges or as a means to re-establish homeostasis after a system is disturbed [75],[76],[77]. An additional characteristic of this mechanism is that it provides a way to transform different levels of input into corresponding durations of signal output and it is this feature that appears to be exploited by cells to interpret graded Shh signalling. This would reconcile the competing models of pattern formation that have emphasized either concentration or time-dependent mechanisms [73]. Furthermore, in contrast to concentration-based mechanisms of morphogen interpretation where concentrations above the saturation limit elicit the same response, a temporal adaptation mechanism allows cells to discriminate between saturating concentrations of ligand (Figure 6B). This would therefore extend the range of concentrations that can be discerned by a cell and shift the functional range to higher concentrations. The effect would be to increase the potential set of responses that can be elicited by a single signal and render the process less susceptible to the higher levels of noise associated with low concentrations of an extrinsic ligand [78]. A trade off with this mechanism, however, is that it requires patterning to occur over a comparatively long time period in which cell position remains stable relative to the gradient. Nevertheless, despite this limitation, it is possible that the interpretation of other morphogens may use similar mechanisms. For example, a correspondence between the activation of target genes and duration of signalling has been noted for Nodal in zebrafish embryos [79] and Dpp in the Drosophila wing disc [80]. In addition, brief exposure to Wg signalling is sufficient to induce low but not high responses in the wing disc [81]. Thus, the elucidation of the mechanism by which Shh provides positional information to neural cells suggests strategies of morphogen interpretation that may be generally applicable in developing tissues.

## Materials and Methods

### Electroporation and Immunocytochemistry

In ovo electroporation, fixation, sectioning of embryos, and immunocytochemistry were performed as described ([21]). Antibodies used were rabbit anti-Arx (a gift from J. Chelly), goat anti-β-galactosidase (Biogenesis), rabbit anti-Chx10 (a gift from T. Jessell), rabbit anti-Cre (Covance), Rabbit anti-Dbx1 [34], mouse anti-En1 (DSHB), mouse anti-Evx1 (DSHB), sheep anti-GFP (Biogenesis), mouse anti-MNR2 (DSHB), rabbit anti-Nkx2.2 (a gift from T. Jessell), mouse anti-Nkx2.2 (DSHB), mouse anti-Nkx6.1 (DSHB), guinea-pig anti-Olig2 [82], mouse anti-Pax6 (DSHB), mouse anti-Pax7 (DSHB), and mouse anti-Shh (DSHB).

### Explants

The open neural plate of HH stage 10 chick embryos was isolated in L-15 (Gibco) media and the intermediate region dissected following Dispase treatment (Gibco). These [i] explants were embedded in Collagen Type I containing DMEM (Gibco). Culture medium contained F-12/Ham (Gibco) supplemented with 2 mM of Glutamine, 50 U/ml of Penicillin, 50 µg/ml of Streptomycin and Mito Serum (BD). Recombinant Shh protein was produced as described [39]. Explants were fixed in 0.1 M phosphate buffer pH7.2 containing 4% PFA prior to immunostaining. Two regions containing approximately 200 cells were chosen from each of 4 or 5 explants for quantitations. For luciferase assays in explants, GBS-luc [83] was electroporated with normalization plasmid (pRL-TK; Promega) 2 h prior to the dissection of the explants [24]. Gli activity was measured using the Dual Luciferase Assay (Promega) and compared to levels of Gli activity in transfected untreated explants.

### In Ovo Cyclopamine Treatment

Eggs were incubated to HH st.18 and windowed. Embryos were treated with 25 µl of 1 mg/mL solution of cyclopamine (Toronto Research Chemicals) in 45% 2-hydropropyl-β-cyclodextrin (HBC; Sigma) [44]. The embryos were re-incubated for a further 24 h and then processed for immunocytochemistry.

### Mouse Lines

The Dbx1-Cre, Olig2-Cre, Shh^Flox^, and *Bcre32* (Tg(Pou3f4-cre)32Cre) mouse lines have been described previously [24],[42],[48],[49] and the conditional lineage tracing alleles engineered in the ROSA26 locus have been described [43],[51]. All studies in mice were carried out with appropriate permissions and in accordance with the Institutional Animal Care and Use Subcommittee of the National Institute for Medical Research.

## Supporting Information

Figure S1 **Representative images of the experiments quantified in Figure 2.** Expression of Evx1 (A–B), En1 (C–D), and Chx10 and MNR2 (E–F) in [i] explants exposed to the indicated concentrations of Shh for 48 h. (G–J) Expression of the indicated markers in [i] explants exposed to 0.5 nM Shh for the indicated periods of time and then transferred to media lacking Shh. All explants were incubated for 48 h prior to fixation and immunostaining.
(2.17 MB TIF)

Figure S2 **Quantification of Pax7 and Dbx1 expression in [i] explants exposed to the indicated concentrations of Shh for 48 h.**
(0.16 MB TIF)

Figure S3 **Cells maintain the competence to respond to Shh and express ventral neural progenitor markers even by generic treatments.** (A; top) Scheme for the experiment. [i] Explants were incubated in media without Shh (grey columns) for 24 h and then exposed to 0.5 nM Shh (blue columns) for the indicated periods of time. The media on some explants (ii)–(iv) was then replaced with fresh media lacking Shh, as indicated, and all explants were analyzed after a total of 48 h ex vivo. Explants were then assayed for the expression of Dbx1, Nkx6.1, and Olig2 (bottom). Quantification of the number of cells expressing the indicated markers in each condition. (B; top) Scheme for the experiment. [i] Explants were incubated with 4 nM Shh (blue columns) or with control media (grey columns) for the indicated periods of time, then harvested (red arrows) and analyzed for the expression of Olig2 and Nkx2.2. (middle and bottom) Quantification of Olig2 and Nkx2.2 in each condition.
(0.62 MB TIF)

Figure S4 **Cells revert to a more dorsal identity when Shh signalling is interrupted.** (A) Scheme for experiments in (B) and (C). Explants were exposed to 0.5 nM Shh (blue columns) for 36 h, either continuously (i) or after 18 h some were transferred to media containing 500 nM cyclopamine (Cyc.) and 0.5 nM Shh (ii; red columns) to block Shh signalling. All explants were assayed 36 h after the start of culture. (B) Representative images of [i] explants exposed to 0.5 nM Shh for 36 h (i) or 0.5 nM Shh for 18 h followed by 500 nM cyclopamine and 0.5 nM Shh for an additional 18 h (ii). Explants assayed for Evx1, En1, Chx10, and MNR2 expression. (C) Quantification of cells expressing Evx1, En1, Chx10, and MNR2 at 36 h in [i] explants cultured continuously for 36 h in 0.5 nM Shh (i) or in conditions (ii) in which signalling is interrupted at 18 h (n≥4; cells/unit ± s.d.). Note that the difference in the number of En1 expressing cells is not significantly different in these experiments, although the trend matches that seen for Evx1 and Chx10. (D) Time course of Dbx1, Nkx6.1, and Olig2 expression. [i] Explants were incubated for 6–36 h with 0.5 nM Shh (solid lines; data as shown in Figure 3B) or 18 h with 0.5 nM Shh followed by 500 nM cyclopamine and 0.5 nM Shh for an additional 6, 12, or 18 h (dashed lines). Quantitation was performed at 6, 12, 18, 24, 30, and 36 h after the start of culture.
(1.17 MB TIF)

Figure S5 **Expression of Shh and Pax6 in the Brn4cre;Shh^flox/flox^ (Shh^CKO^) mice.** The expression analysis was done on the same embryos as in Figure 5F, 5G (A, B). At e10.5 mutant embryos (Shh^CKO^) had lost Shh expression in the neural tube (B) but not the notochord (arrowhead). (C, D) A ventral expansion in the domain of cells expressing Pax6 (D) compared to control littermates (C) (double-headed arrow indicates distances from the floor plate to the ventral border of Pax6 expression). Notably, the notochord had regressed from the neural tube in Shh^CKO^ embryos.
(0.69 MB TIF)

## Abbreviations

DV: dorsal-ventral
HH: Hamburger and Hamilton
Hh: Hedgehog
MNs: motor neurons
Shh: Sonic hedgehog
TGF-β: Transforming Growth Factor beta
Wnt: Wingless
