# Supplementary figures and images for: Dynamic Assignment and Maintenance of Positional Identity in the Ventral Neural Tube by the Morphogen Sonic Hedgehog
Source: PLoS Biol. 2010 Jun 1;8(6):e1000382. doi: 10.1371/journal.pbio.1000382 (PMC2879390; doi:10.1371/journal.pbio.1000382)

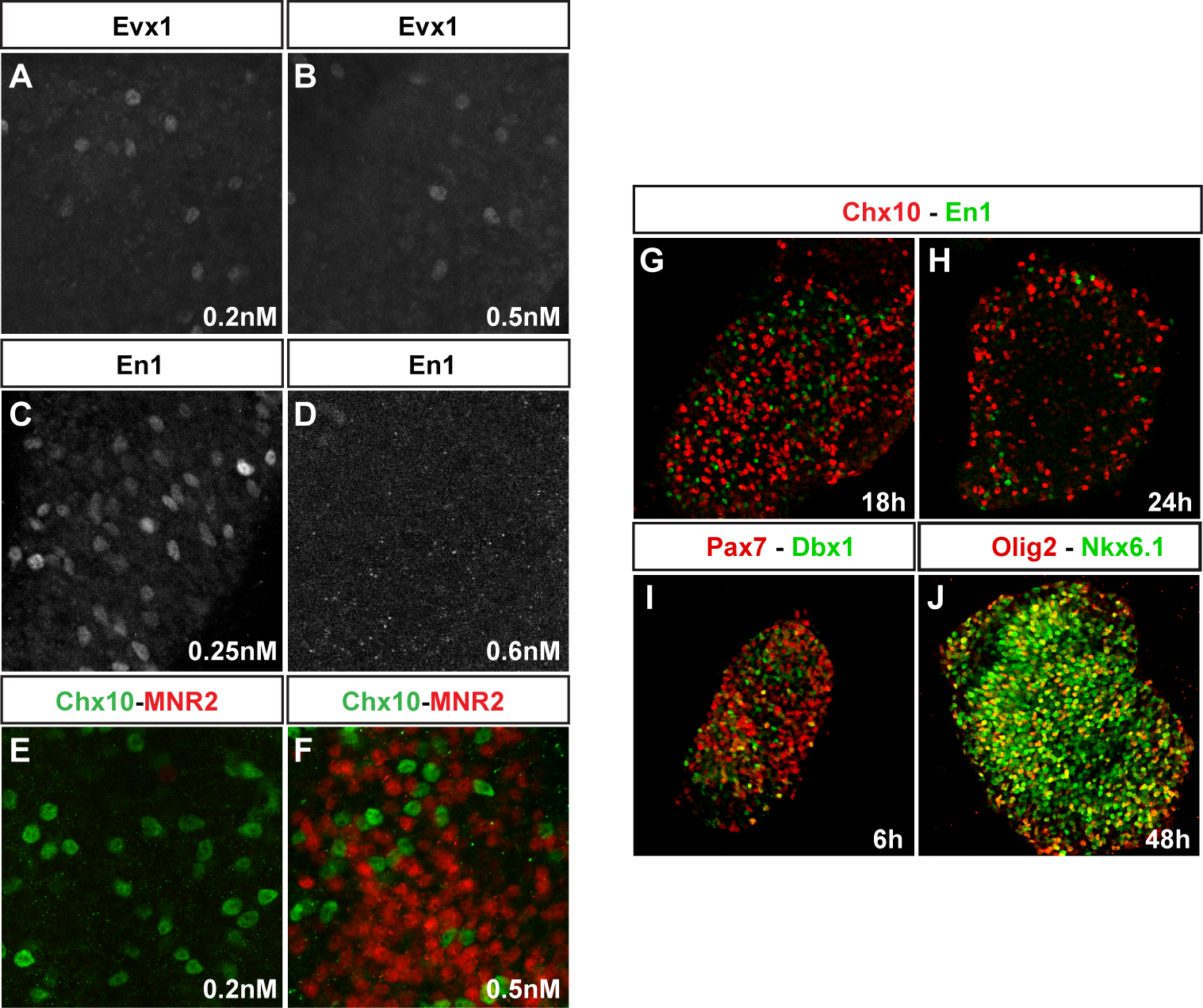

Supplement: Figure S1 — Representative images of the experiments quantified in Figure 2. Expression of Evx1 (A–B), En1 (C–D), and Chx10 and MNR2 (E–F) in [i] explants exposed to the indicated concentrations of Shh for 48 h. (G–J) Expression of the indicated markers in [i] explants exposed to 0.5 nM Shh for the indicated periods of time and then transferred to media lacking Shh. All explants were incubated for 48 h prior to fixation and immunostaining. (2.17 MB TIF) [file pbio.1000382.s001.tif]

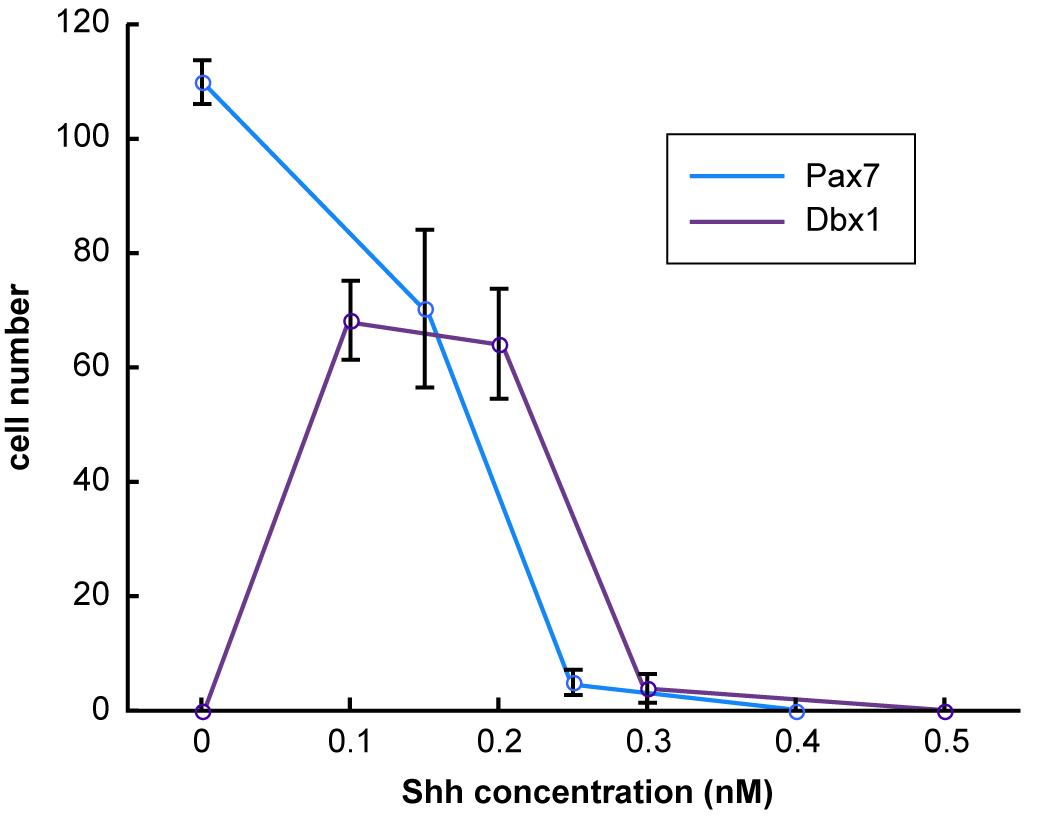

Supplement: Figure S2 — Quantification of Pax7 and Dbx1 expression in [i] explants exposed to the indicated concentrations of Shh for 48 h. (0.16 MB TIF) [file pbio.1000382.s002.tif]

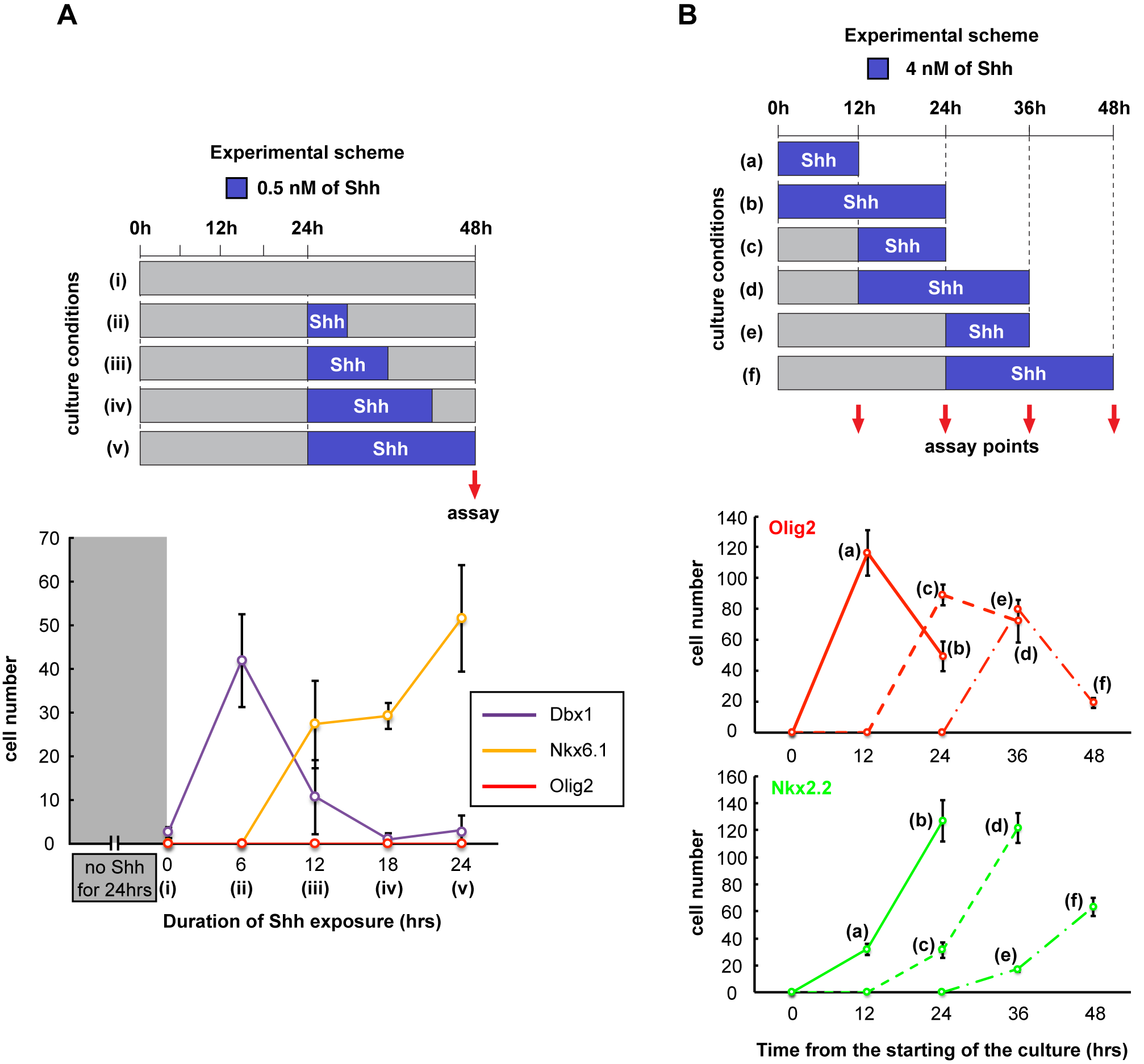

Supplement: Figure S3 — Cells maintain the competence to respond to Shh and express ventral neural progenitor markers even by generic treatments. (A; top) Scheme for the experiment. [i] Explants were incubated in media without Shh (grey columns) for 24 h and then exposed to 0.5 nM Shh (blue columns) for the indicated periods of time. The media on some explants (ii)–(iv) was then replaced with fresh media lacking Shh, as indicated, and all explants were analyzed after a total of 48 h ex vivo. Explants were then assayed for the expression of Dbx1, Nkx6.1, and Olig2 (bottom). Quantification of the number of cells expressing the indicated markers in each condition. (B; top) Scheme for the experiment. [i] Explants were incubated with 4 nM Shh (blue columns) or with control media (grey columns) for the indicated periods of time, then harvested (red arrows) and analyzed for the expression of Olig2 and Nkx2.2. (middle and bottom) Quantification of Olig2 and Nkx2.2 in each condition. (0.62 MB TIF) [file pbio.1000382.s003.tif]

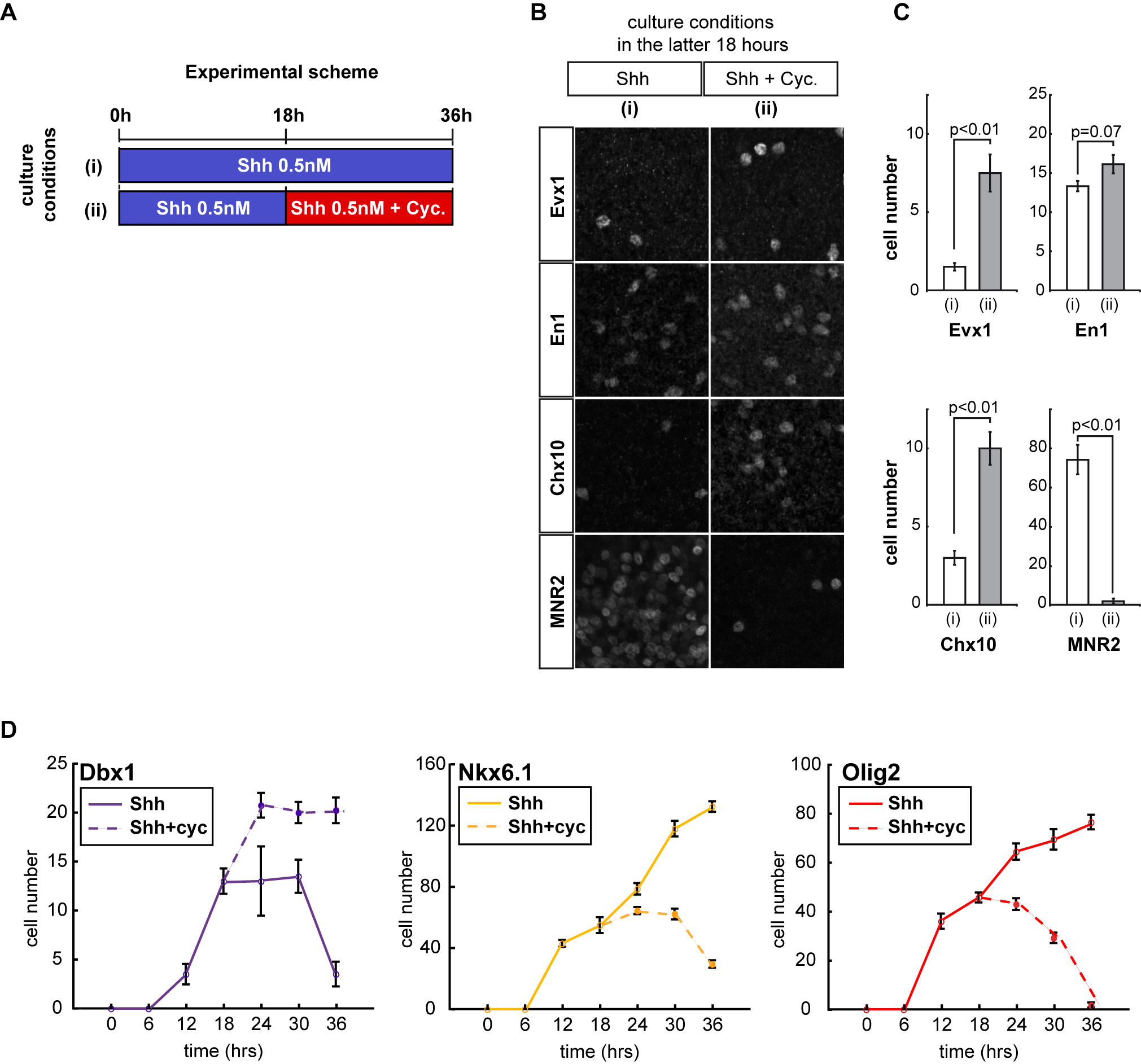

Supplement: Figure S4 — Cells revert to a more dorsal identity when Shh signalling is interrupted. (A) Scheme for experiments in (B) and (C). Explants were exposed to 0.5 nM Shh (blue columns) for 36 h, either continuously (i) or after 18 h some were transferred to media containing 500 nM cyclopamine (Cyc.) and 0.5 nM Shh (ii; red columns) to block Shh signalling. All explants were assayed 36 h after the start of culture. (B) Representative images of [i] explants exposed to 0.5 nM Shh for 36 h (i) or 0.5 nM Shh for 18 h followed by 500 nM cyclopamine and 0.5 nM Shh for an additional 18 h (ii). Explants assayed for Evx1, En1, Chx10, and MNR2 expression. (C) Quantification of cells expressing Evx1, En1, Chx10, and MNR2 at 36 h in [i] explants cultured continuously for 36 h in 0.5 nM Shh (i) or in conditions (ii) in which signalling is interrupted at 18 h (n≥4; cells/unit ± s.d.). Note that the difference in the number of En1 expressing cells is not significantly different in these experiments, although the trend matches that seen for Evx1 and Chx10. (D) Time course of Dbx1, Nkx6.1, and Olig2 expression. [i] Explants were incubated for 6–36 h with 0.5 nM Shh (solid lines; data as shown in Figure 3B) or 18 h with 0.5 nM Shh followed by 500 nM cyclopamine and 0.5 nM Shh for an additional 6, 12, or 18 h (dashed lines). Quantitation was performed at 6, 12, 18, 24, 30, and 36 h after the start of culture. (1.17 MB TIF) [file pbio.1000382.s004.tif]

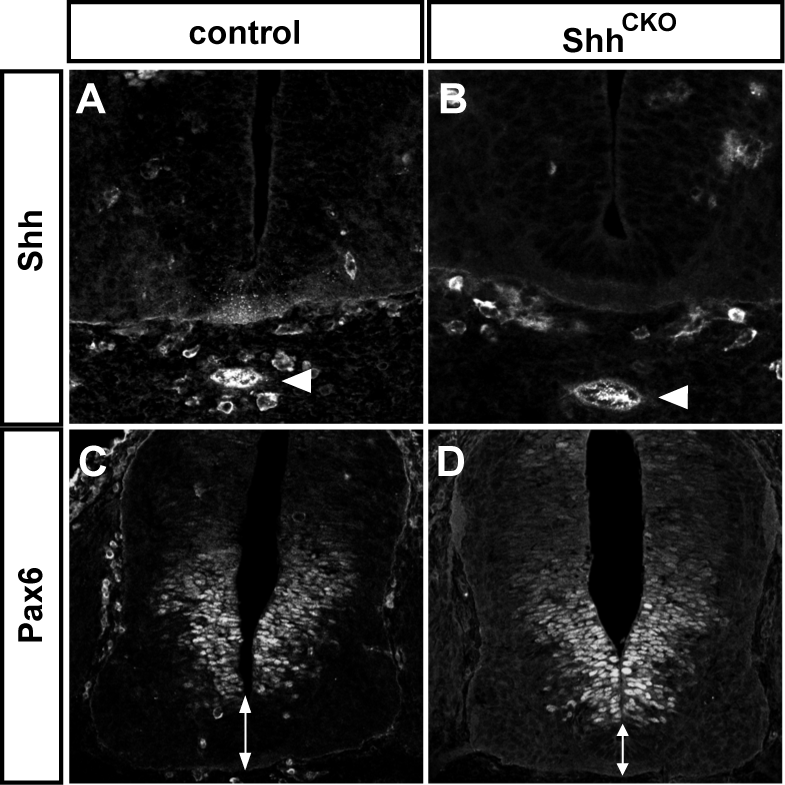

Supplement: Figure S5 — Expression of Shh and Pax6 in the Brn4cre;Shhflox/flox (ShhCKO) mice. The expression analysis was done on the same embryos as in Figure 5F, 5G (A, B). At e10.5 mutant embryos (ShhCKO) had lost Shh expression in the neural tube (B) but not the notochord (arrowhead). (C, D) A ventral expansion in the domain of cells expressing Pax6 (D) compared to control littermates (C) (double-headed arrow indicates distances from the floor plate to the ventral border of Pax6 expression). Notably, the notochord had regressed from the neural tube in ShhCKO embryos. (0.69 MB TIF) [file pbio.1000382.s005.tif]
